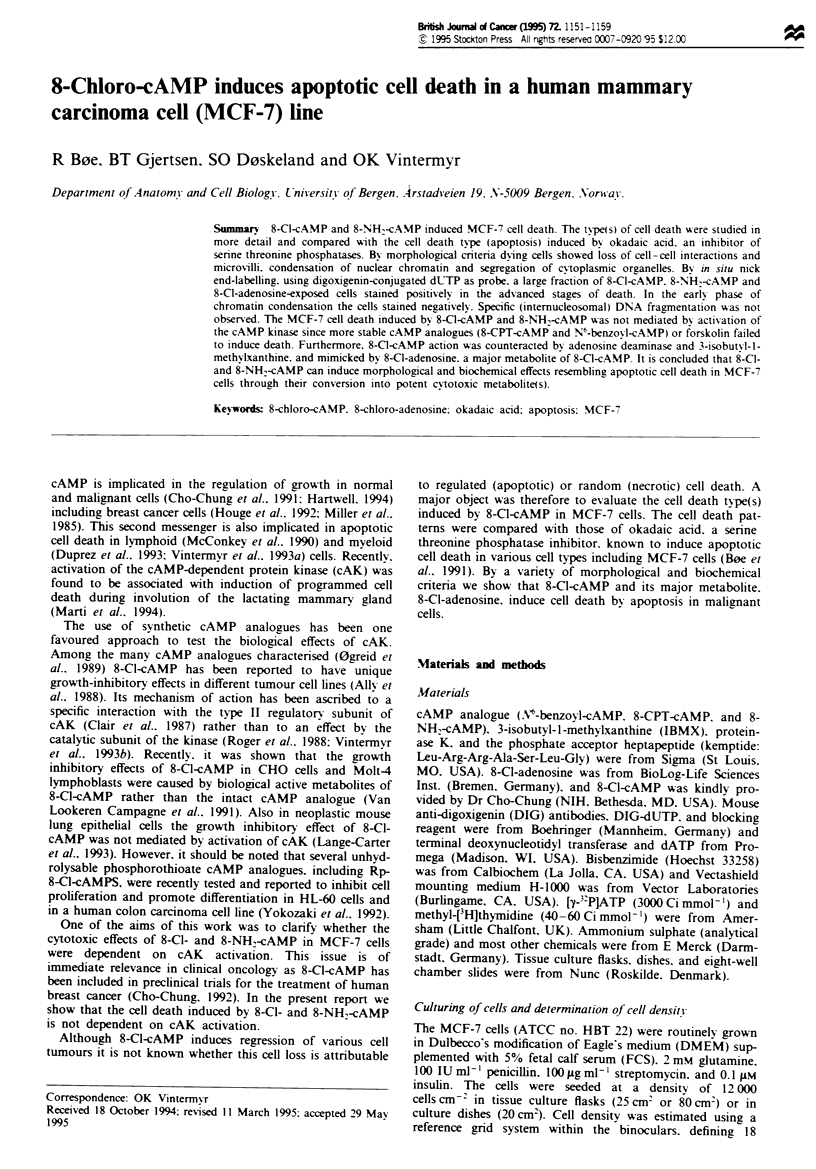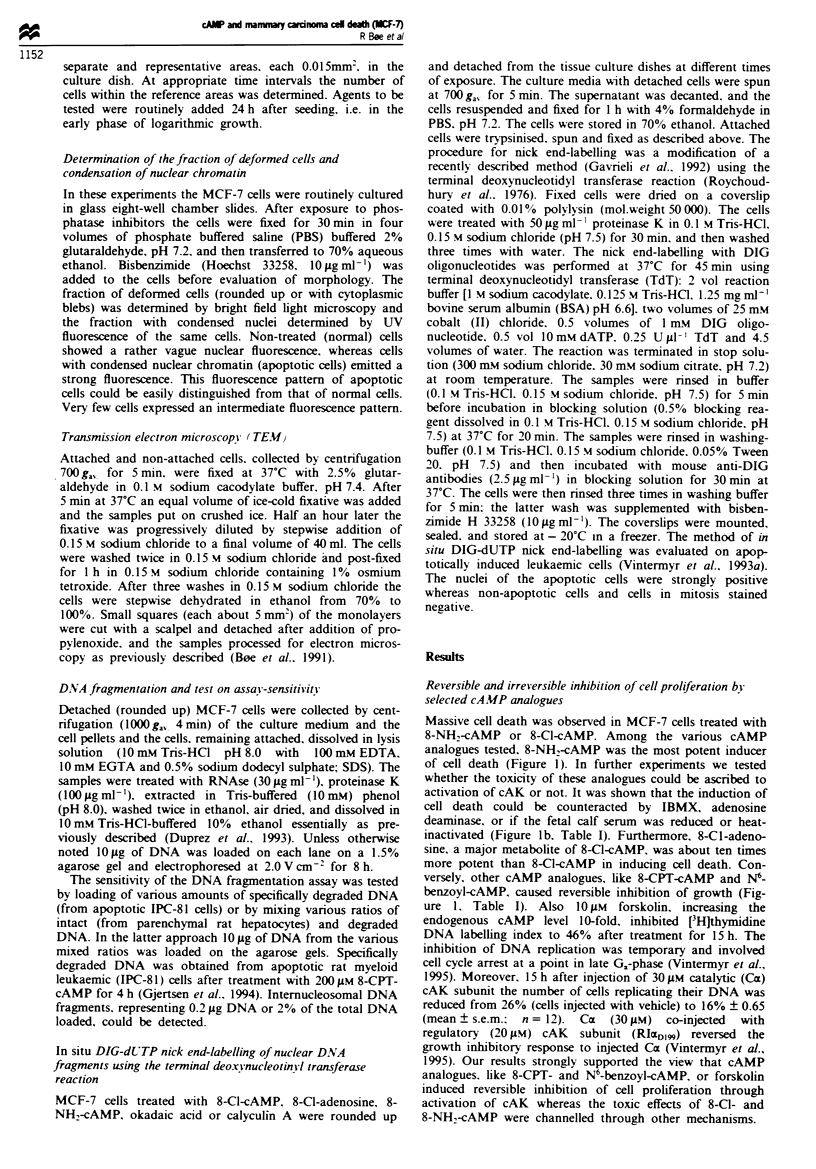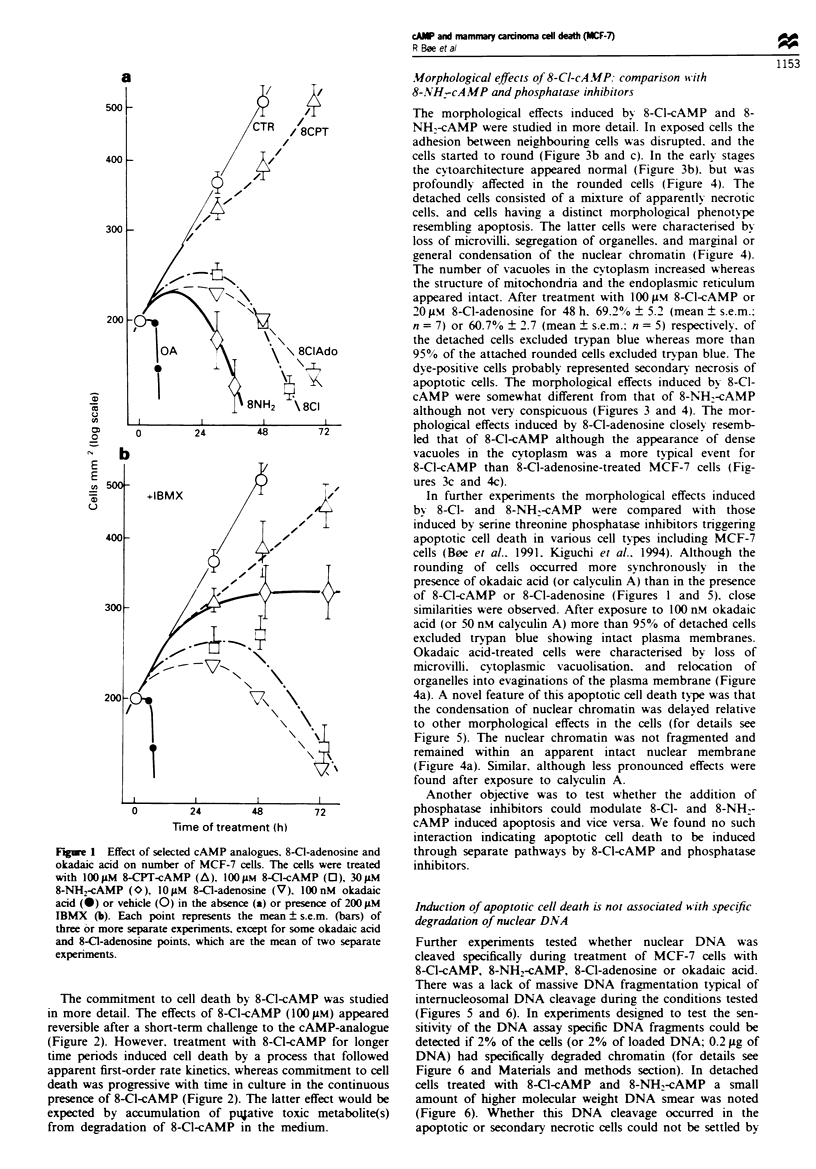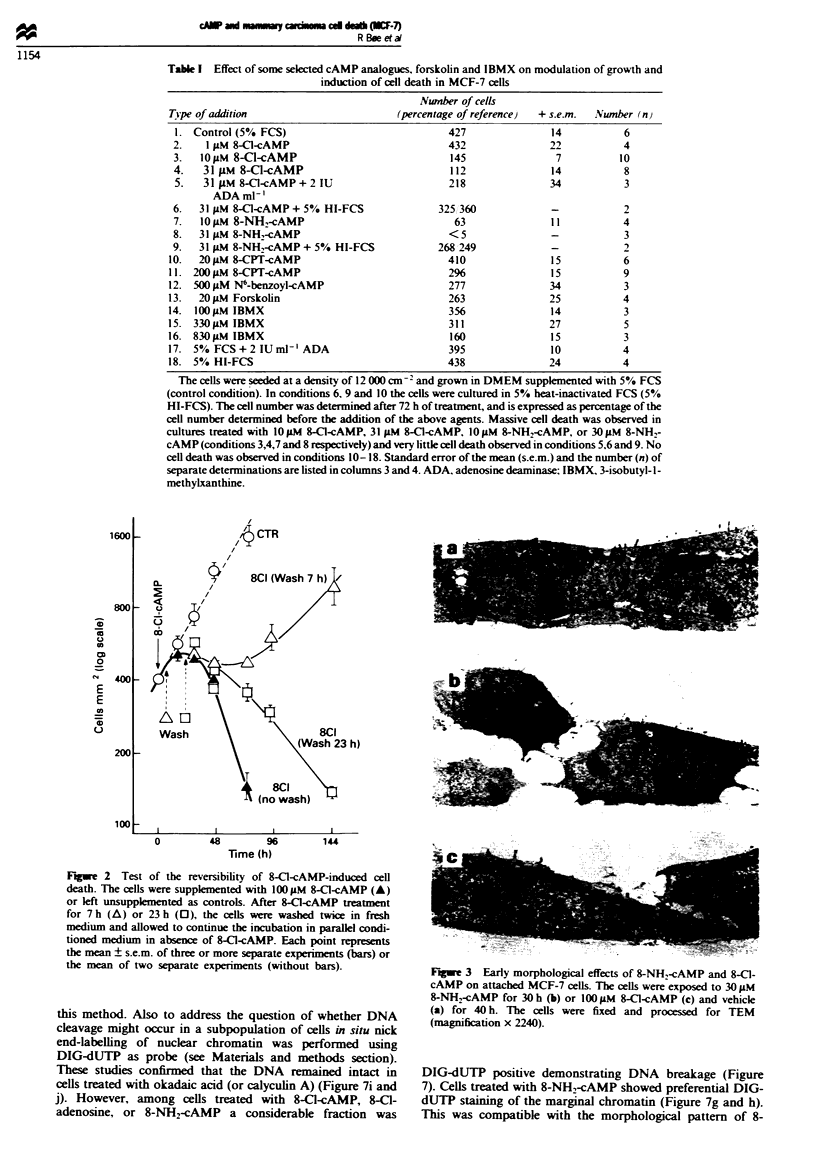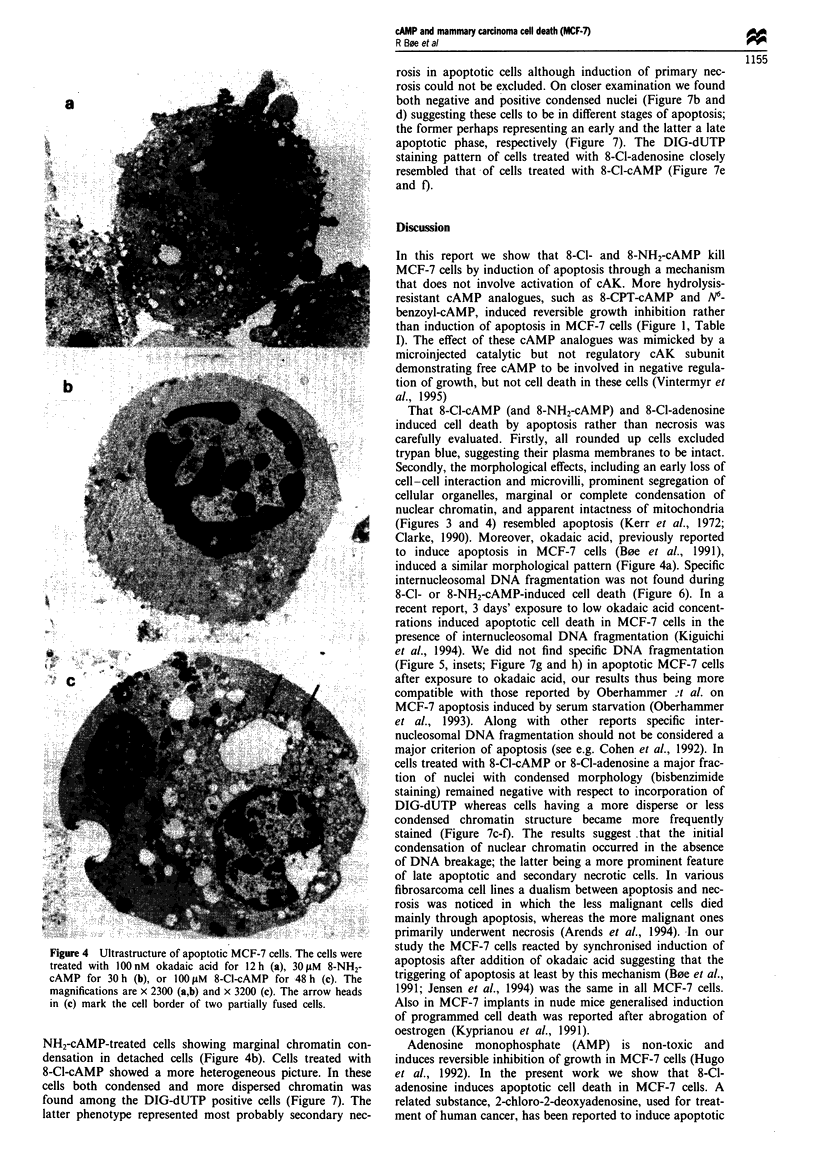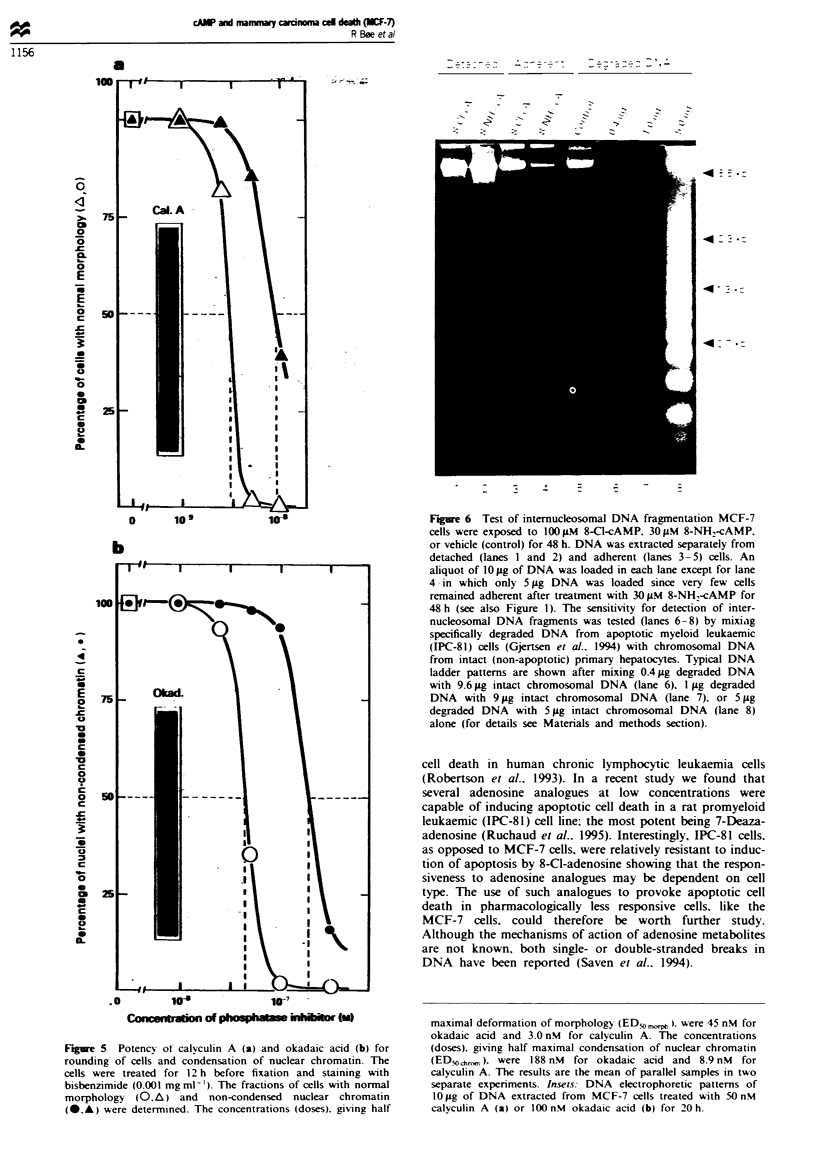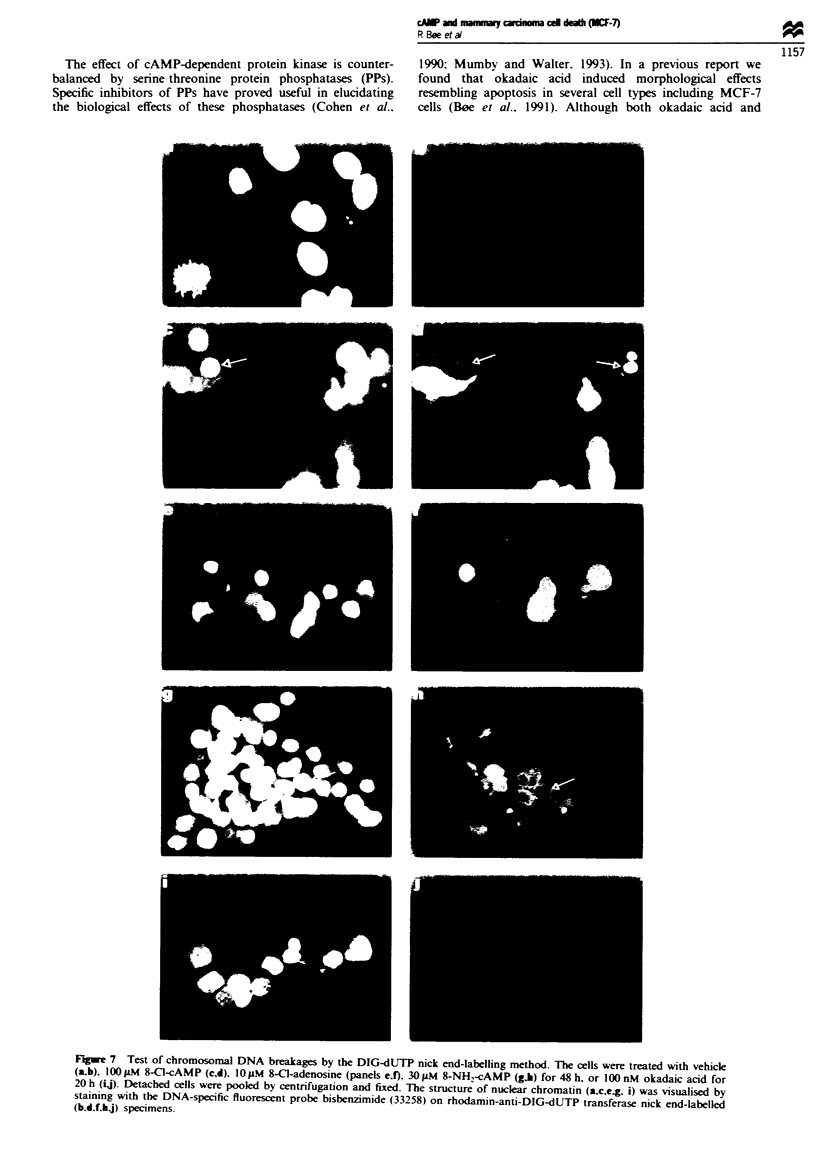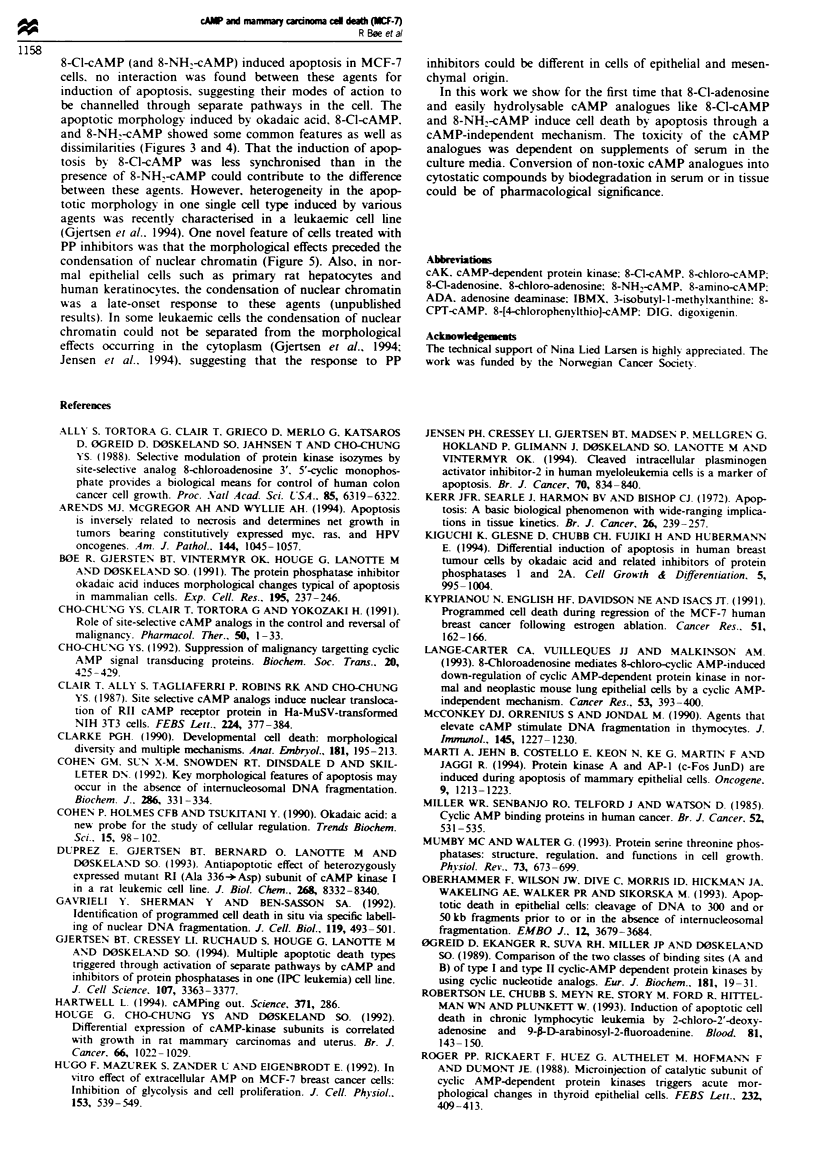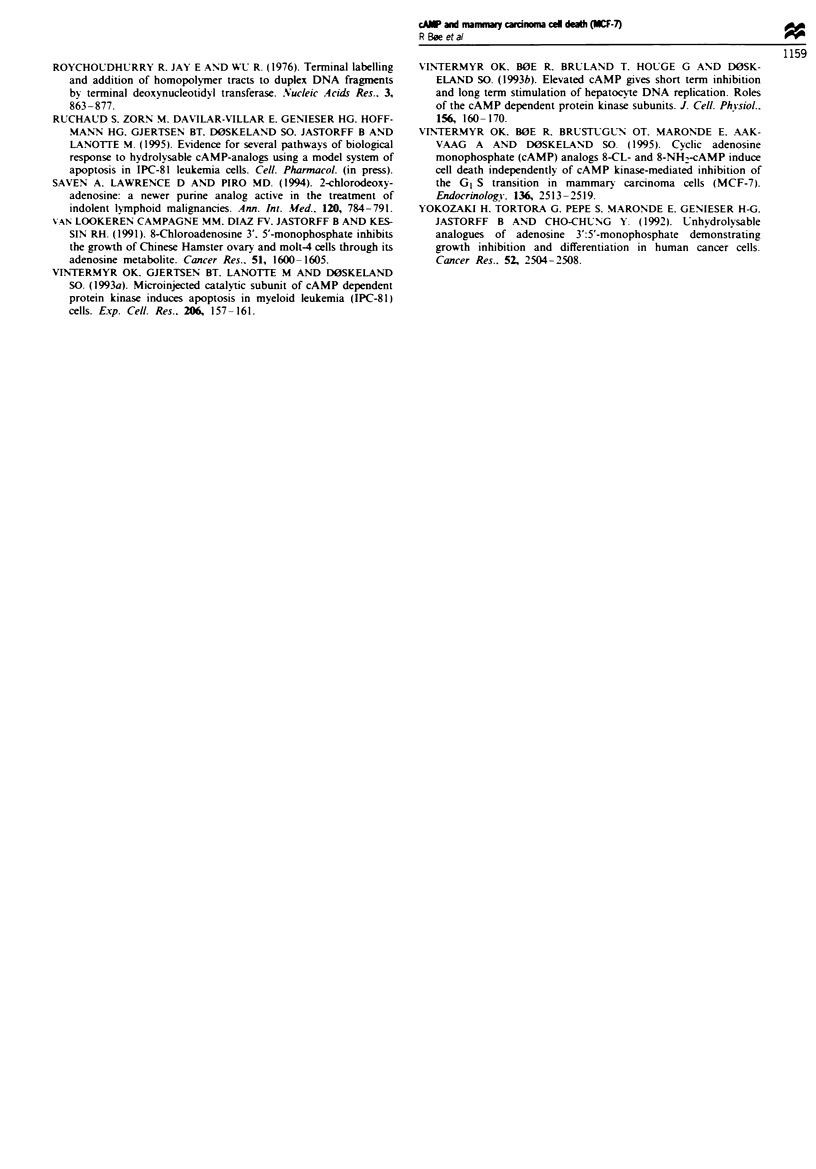# 8-Chloro-cAMP induces apoptotic cell death in a human mammary carcinoma cell (MCF-7) line.

**DOI:** 10.1038/bjc.1995.479

**Published:** 1995-11

**Authors:** R. Bøe, B. T. Gjertsen, S. O. Døskeland, O. K. Vintermyr

**Affiliations:** Department of Anatomy and Cell Biology, University of Bergen, Norway.

## Abstract

**Images:**


British Journal of Cancer (1995) 72, 1151–1159

© 1995 Stockton Press All rights reserved 0007-0920/95 $12.00

### 8-Chloro-cAMP induces apoptotic cell death in a human mammary carcinoma cell (MCF-7) line

R Bøe, BT Gjertsen, SO Døskeland and OK Vintermyr

*Department of Anatomy and Cell Biology, University of Bergen, Årstadveien 19, N-5009 Bergen, Norway.*

**Summary** 8-Cl-cAMP and 8-$NH_2$-cAMP induced MCF-7 cell death. The type(s) of cell death were studied in more detail and compared with the cell death type (apoptosis) induced by okadaic acid, an inhibitor of serine threonine phosphatases. By morphological criteria dying cells showed loss of cell–cell interactions and microvilli, condensation of nuclear chromatin and segregation of cytoplasmic organelles. By *in situ* nick end-labelling, using digoxigenin-conjugated dUTP as probe, a large fraction of 8-Cl-cAMP, 8-$NH_2$-cAMP and 8-Cl-adenosine-exposed cells stained positively in the advanced stages of death. In the early phase of chromatin condensation the cells stained negatively. Specific (internucleosomal) DNA fragmentation was not observed. The MCF-7 cell death induced by 8-Cl-cAMP and 8-$NH_2$-cAMP was not mediated by activation of the cAMP kinase since more stable cAMP analogues (8-CPT-cAMP and $N^6$-benzoyl-cAMP) or forskolin failed to induce death. Furthermore, 8-Cl-cAMP action was counteracted by adenosine deaminase and 3-isobutyl-1-methylxanthine, and mimicked by 8-Cl-adenosine, a major metabolite of 8-Cl-cAMP. It is concluded that 8-Cl- and 8-$NH_2$-cAMP can induce morphological and biochemical effects resembling apoptotic cell death in MCF-7 cells through their conversion into potent cytotoxic metabolite(s).

**Keywords:** 8-chloro-cAMP, 8-chloro-adenosine; okadaic acid; apoptosis; MCF-7

cAMP is implicated in the regulation of growth in normal and malignant cells (Cho-Chung *et al.*, 1991; Hartwell, 1994) including breast cancer cells (Houge *et al.*, 1992; Miller *et al.*, 1985). This second messenger is also implicated in apoptotic cell death in lymphoid (McConkey *et al.*, 1990) and myeloid (Duprez *et al.*, 1993; Vintermyr *et al.*, 1993*a*) cells. Recently, activation of the cAMP-dependent protein kinase (cAK) was found to be associated with induction of programmed cell death during involution of the lactating mammary gland (Marti *et al.*, 1994).

The use of synthetic cAMP analogues has been one favoured approach to test the biological effects of cAK. Among the many cAMP analogues characterised (Øgreid *et al.*, 1989) 8-Cl-cAMP has been reported to have unique growth-inhibitory effects in different tumour cell lines (Ally *et al.*, 1988). Its mechanism of action has been ascribed to a specific interaction with the type II regulatory subunit of cAK (Clair *et al.*, 1987) rather than to an effect by the catalytic subunit of the kinase (Roger *et al.*, 1988; Vintermyr *et al.*, 1993*b*). Recently, it was shown that the growth inhibitory effects of 8-Cl-cAMP in CHO cells and Molt-4 lymphoblasts were caused by biological active metabolites of 8-Cl-cAMP rather than the intact cAMP analogue (Van Lookeren Campagne *et al.*, 1991). Also in neoplastic mouse lung epithelial cells the growth inhibitory effect of 8-Cl-cAMP was not mediated by activation of cAK (Lange-Carter *et al.*, 1993). However, it should be noted that several unhydrolysable phosphorothioate cAMP analogues, including Rp-8-Cl-cAMPS, were recently tested and reported to inhibit cell proliferation and promote differentiation in HL-60 cells and in a human colon carcinoma cell line (Yokozaki *et al.*, 1992).

One of the aims of this work was to clarify whether the cytotoxic effects of 8-Cl- and 8-$NH_2$-cAMP in MCF-7 cells were dependent on cAK activation. This issue is of immediate relevance in clinical oncology as 8-Cl-cAMP has been included in preclinical trials for the treatment of human breast cancer (Cho-Chung, 1992). In the present report we show that the cell death induced by 8-Cl- and 8-$NH_2$-cAMP is not dependent on cAK activation.

Although 8-Cl-cAMP induces regression of various cell tumours it is not known whether this cell loss is attributable to regulated (apoptotic) or random (necrotic) cell death. A major object was therefore to evaluate the cell death type(s) induced by 8-Cl-cAMP in MCF-7 cells. The cell death patterns were compared with those of okadaic acid, a serine threonine phosphatase inhibitor, known to induce apoptotic cell death in various cell types including MCF-7 cells (Bøe *et al.*, 1991). By a variety of morphological and biochemical criteria we show that 8-Cl-cAMP and its major metabolite, 8-Cl-adenosine, induce cell death by apoptosis in malignant cells.

Correspondence: OK Vintermyr
Received 18 October 1994; revised 11 March 1995; accepted 29 May 1995

### Materials and methods

### *Materials*

cAMP analogue ($N^6$-benzoyl-cAMP, 8-CPT-cAMP, and 8-$NH_2$-cAMP), 3-isobutyl-1-methylxanthine (IBMX), proteinase K, and the phosphate acceptor heptapeptide (kemptide: Leu-Arg-Arg-Ala-Ser-Leu-Gly) were from Sigma (St Louis, MO, USA). 8-Cl-adenosine was from BioLog-Life Sciences Inst. (Bremen, Germany), and 8-Cl-cAMP was kindly provided by Dr Cho-Chung (NIH, Bethesda, MD, USA). Mouse anti-digoxigenin (DIG) antibodies, DIG-dUTP, and blocking reagent were from Boehringer (Mannheim, Germany) and terminal deoxynucleotidyl transferase and dATP from Promega (Madison, WI, USA). Bisbenzimide (Hoechst 33258) was from Calbiochem (La Jolla, CA, USA) and Vectashield mounting medium H-1000 was from Vector Laboratories (Burlingame, CA, USA). [$\gamma$-$^{32}$P]ATP (3000 Ci mmol$^{-1}$) and methyl-[$^3$H]thymidine (40–60 Ci mmol$^{-1}$) were from Amersham (Little Chalfont, UK). Ammonium sulphate (analytical grade) and most other chemicals were from E Merck (Darmstadt, Germany). Tissue culture flasks, dishes, and eight-well chamber slides were from Nunc (Roskilde, Denmark).

### *Culturing of cells and determination of cell density*

The MCF-7 cells (ATCC no. HBT 22) were routinely grown in Dulbecco's modification of Eagle's medium (DMEM) supplemented with 5% fetal calf serum (FCS), 2 mM glutamine, 100 IU ml$^{-1}$ penicillin, 100 $\mu$g ml$^{-1}$ streptomycin, and 0.1 $\mu$M insulin. The cells were seeded at a density of 12 000 cells cm$^{-2}$ in tissue culture flasks (25 cm$^2$ or 80 cm$^2$) or in culture dishes (20 cm$^2$). Cell density was estimated using a reference grid system within the binoculars, defining 18

separate and representative areas, each 0.015mm², in the culture dish. At appropriate time intervals the number of cells within the reference areas was determined. Agents to be tested were routinely added 24 h after seeding, i.e. in the early phase of logarithmic growth.

*Determination of the fraction of deformed cells and condensation of nuclear chromatin*

In these experiments the MCF-7 cells were routinely cultured in glass eight-well chamber slides. After exposure to phosphatase inhibitors the cells were fixed for 30 min in four volumes of phosphate buffered saline (PBS) buffered 2% glutaraldehyde, pH 7.2, and then transferred to 70% aqueous ethanol. Bisbenzimide (Hoechst 33258, 10 μg ml$^{-1}$) was added to the cells before evaluation of morphology. The fraction of deformed cells (rounded up or with cytoplasmic blebs) was determined by bright field light microscopy and the fraction with condensed nuclei determined by UV fluorescence of the same cells. Non-treated (normal) cells showed a rather vague nuclear fluorescence, whereas cells with condensed nuclear chromatin (apoptotic cells) emitted a strong fluorescence. This fluorescence pattern of apoptotic cells could be easily distinguished from that of normal cells. Very few cells expressed an intermediate fluorescence pattern.

*Transmission electron microscopy (TEM)*

Attached and non-attached cells, collected by centrifugation 700 $g_{av}$ for 5 min, were fixed at 37°C with 2.5% glutaraldehyde in 0.1 M sodium cacodylate buffer, pH 7.4. After 5 min at 37°C an equal volume of ice-cold fixative was added and the samples put on crushed ice. Half an hour later the fixative was progressively diluted by stepwise addition of 0.15 M sodium chloride to a final volume of 40 ml. The cells were washed twice in 0.15 M sodium chloride and post-fixed for 1 h in 0.15 M sodium chloride containing 1% osmium tetroxide. After three washes in 0.15 M sodium chloride the cells were stepwise dehydrated in ethanol from 70% to 100%. Small squares (each about 5 mm²) of the monolayers were cut with a scalpel and detached after addition of propylenoxide, and the samples processed for electron microscopy as previously described (Bøe *et al.*, 1991).

*DNA fragmentation and test on assay-sensitivity*

Detached (rounded up) MCF-7 cells were collected by centrifugation (1000 $g_{av}$ 4 min) of the culture medium and the cell pellets and the cells, remaining attached, dissolved in lysis solution (10 mM Tris-HCl pH 8.0 with 100 mM EDTA, 10 mM EGTA and 0.5% sodium dodecyl sulphate; SDS). The samples were treated with RNAse (30 μg ml$^{-1}$), proteinase K (100 μg ml$^{-1}$), extracted in Tris-buffered (10 mM) phenol (pH 8.0), washed twice in ethanol, air dried, and dissolved in 10 mM Tris-HCl-buffered 10% ethanol essentially as previously described (Duprez *et al.*, 1993). Unless otherwise noted 10 μg of DNA was loaded on each lane on a 1.5% agarose gel and electrophoresed at 2.0 V cm$^{-2}$ for 8 h.

The sensitivity of the DNA fragmentation assay was tested by loading of various amounts of specifically degraded DNA (from apoptotic IPC-81 cells) or by mixing various ratios of intact (from parenchymal rat hepatocytes) and degraded DNA. In the latter approach 10 μg of DNA from the various mixed ratios was loaded on the agarose gels. Specifically degraded DNA was obtained from apoptotic rat myeloid leukaemic (IPC-81) cells after treatment with 200 μM 8-CPT-cAMP for 4 h (Gjertsen *et al.*, 1994). Internucleosomal DNA fragments, representing 0.2 μg DNA or 2% of the total DNA loaded, could be detected.

In situ *DIG-dUTP nick end-labelling of nuclear DNA fragments using the terminal deoxynucleotinyl transferase reaction*

MCF-7 cells treated with 8-Cl-cAMP, 8-Cl-adenosine, 8-$NH_2$-cAMP, okadaic acid or calyculin A were rounded up and detached from the tissue culture dishes at different times of exposure. The culture media with detached cells were spun at 700 $g_{av}$ for 5 min. The supernatant was decanted, and the cells resuspended and fixed for 1 h with 4% formaldehyde in PBS, pH 7.2. The cells were stored in 70% ethanol. Attached cells were trypsinised, spun and fixed as described above. The procedure for nick end-labelling was a modification of a recently described method (Gavrieli *et al.*, 1992) using the terminal deoxynucleotidyl transferase reaction (Roychoudhury *et al.*, 1976). Fixed cells were dried on a coverslip coated with 0.01% polylysin (mol.weight 50 000). The cells were treated with 50 μg ml$^{-1}$ proteinase K in 0.1 M Tris-HCl, 0.15 M sodium chloride (pH 7.5) for 30 min, and then washed three times with water. The nick end-labelling with DIG oligonucleotides was performed at 37°C for 45 min using terminal deoxynucleotidyl transferase (TdT): 2 vol reaction buffer [1 M sodium cacodylate, 0.125 M Tris-HCl, 1.25 mg ml$^{-1}$ bovine serum albumin (BSA) pH 6.6], two volumes of 25 mM cobalt (II) chloride, 0.5 volumes of 1 mM DIG oligonucleotide, 0.5 vol 10 mM dATP, 0.25 U μl$^{-1}$ TdT and 4.5 volumes of water. The reaction was terminated in stop solution (300 mM sodium chloride, 30 mM sodium citrate, pH 7.2) at room temperature. The samples were rinsed in buffer (0.1 M Tris-HCl, 0.15 M sodium chloride, pH 7.5) for 5 min before incubation in blocking solution (0.5% blocking reagent dissolved in 0.1 M Tris-HCl, 0.15 M sodium chloride, pH 7.5) at 37°C for 20 min. The samples were rinsed in washing-buffer (0.1 M Tris-HCl, 0.15 M sodium chloride, 0.05% Tween 20, pH 7.5) and then incubated with mouse anti-DIG antibodies (2.5 μg ml$^{-1}$) in blocking solution for 30 min at 37°C. The cells were then rinsed three times in washing buffer for 5 min; the latter wash was supplemented with bisbenzimide H 33258 (10 μg ml$^{-1}$). The coverslips were mounted, sealed, and stored at − 20°C in a freezer. The method of *in situ* DIG-dUTP nick end-labelling was evaluated on apoptotically induced leukaemic cells (Vintermyr *et al.*, 1993*a*). The nuclei of the apoptotic cells were strongly positive whereas non-apoptotic cells and cells in mitosis stained negative.

### Results

*Reversible and irreversible inhibition of cell proliferation by selected cAMP analogues*

Massive cell death was observed in MCF-7 cells treated with 8-$NH_2$-cAMP or 8-Cl-cAMP. Among the various cAMP analogues tested, 8-$NH_2$-cAMP was the most potent inducer of cell death (Figure 1). In further experiments we tested whether the toxicity of these analogues could be ascribed to activation of cAK or not. It was shown that the induction of cell death could be counteracted by IBMX, adenosine deaminase, or if the fetal calf serum was reduced or heat-inactivated (Figure 1b, Table I). Furthermore, 8-Cl-adenosine, a major metabolite of 8-Cl-cAMP, was about ten times more potent than 8-Cl-cAMP in inducing cell death. Conversely, other cAMP analogues, like 8-CPT-cAMP and $N^6$-benzoyl-cAMP, caused reversible inhibition of growth (Figure 1, Table I). Also 10 μM forskolin, increasing the endogenous cAMP level 10-fold, inhibited [$^3$H]thymidine DNA labelling index to 46% after treatment for 15 h. The inhibition of DNA replication was temporary and involved cell cycle arrest at a point in late $G_x$-phase (Vintermyr *et al.*, 1995). Moreover, 15 h after injection of 30 μM catalytic (Cα) cAK subunit the number of cells replicating their DNA was reduced from 26% (cells injected with vehicle) to 16% ± 0.65 (mean ± s.e.m.; $n = 12$). Cα (30 μM) co-injected with regulatory (20 μM) cAK subunit ($RI\alpha_{D199}$) reversed the growth inhibitory response to injected Cα (Vintermyr *et al.*, 1995). Our results strongly supported the view that cAMP analogues, like 8-CPT- and $N^6$-benzoyl-cAMP, or forskolin induced reversible inhibition of cell proliferation through activation of cAK whereas the toxic effects of 8-Cl- and 8-$NH_2$-cAMP were channelled through other mechanisms.

**Figure 1** Effect of selected cAMP analogues, 8-Cl-adenosine and okadaic acid on number of MCF-7 cells. The cells were treated with 100 µM 8-CPT-cAMP (△), 100 µM 8-Cl-cAMP (□), 30 µM $8\text{-}NH_2$-cAMP (◇), 10 µM 8-Cl-adenosine (▽), 100 nM okadaic acid (●) or vehicle (○) in the absence (**a**) or presence of 200 µM IBMX (**b**). Each point represents the mean ± s.e.m. (bars) of three or more separate experiments, except for some okadaic acid and 8-Cl-adenosine points, which are the mean of two separate experiments.

The commitment to cell death by 8-Cl-cAMP was studied in more detail. The effects of 8-Cl-cAMP (100 µM) appeared reversible after a short-term challenge to the cAMP-analogue (Figure 2). However, treatment with 8-Cl-cAMP for longer time periods induced cell death by a process that followed apparent first-order rate kinetics, whereas commitment to cell death was progressive with time in culture in the continuous presence of 8-Cl-cAMP (Figure 2). The latter effect would be expected by accumulation of putative toxic metabolite(s) from degradation of 8-Cl-cAMP in the medium.

*Morphological effects of 8-Cl-cAMP: comparison with $8\text{-}NH_2$-cAMP and phosphatase inhibitors*

The morphological effects induced by 8-Cl-cAMP and $8\text{-}NH_2$-cAMP were studied in more detail. In exposed cells the adhesion between neighbouring cells was disrupted, and the cells started to round (Figure 3b and c). In the early stages the cytoarchitecture appeared normal (Figure 3b), but was profoundly affected in the rounded cells (Figure 4). The detached cells consisted of a mixture of apparently necrotic cells, and cells having a distinct morphological phenotype resembling apoptosis. The latter cells were characterised by loss of microvilli, segregation of organelles, and marginal or general condensation of the nuclear chromatin (Figure 4). The number of vacuoles in the cytoplasm increased whereas the structure of mitochondria and the endoplasmic reticulum appeared intact. After treatment with 100 µM 8-Cl-cAMP or 20 µM 8-Cl-adenosine for 48 h, 69.2% ± 5.2 (mean ± s.e.m.; $n = 7$) or 60.7% ± 2.7 (mean ± s.e.m.; $n = 5$) respectively, of the detached cells excluded trypan blue whereas more than 95% of the attached rounded cells excluded trypan blue. The dye-positive cells probably represented secondary necrosis of apoptotic cells. The morphological effects induced by 8-Cl-cAMP were somewhat different from that of $8\text{-}NH_2$-cAMP although not very conspicuous (Figures 3 and 4). The morphological effects induced by 8-Cl-adenosine closely resembled that of 8-Cl-cAMP although the appearance of dense vacuoles in the cytoplasm was a more typical event for 8-Cl-cAMP than 8-Cl-adenosine-treated MCF-7 cells (Figures 3c and 4c).

In further experiments the morphological effects induced by 8-Cl- and $8\text{-}NH_2$-cAMP were compared with those induced by serine threonine phosphatase inhibitors triggering apoptotic cell death in various cell types including MCF-7 cells (Bøe *et al*., 1991, Kiguchi *et al*., 1994). Although the rounding of cells occurred more synchronously in the presence of okadaic acid (or calyculin A) than in the presence of 8-Cl-cAMP or 8-Cl-adenosine (Figures 1 and 5), close similarities were observed. After exposure to 100 nM okadaic acid (or 50 nM calyculin A) more than 95% of detached cells excluded trypan blue showing intact plasma membranes. Okadaic acid-treated cells were characterised by loss of microvilli, cytoplasmic vacuolisation, and relocation of organelles into evaginations of the plasma membrane (Figure 4a). A novel feature of this apoptotic cell death type was that the condensation of nuclear chromatin was delayed relative to other morphological effects in the cells (for details see Figure 5). The nuclear chromatin was not fragmented and remained within an apparent intact nuclear membrane (Figure 4a). Similar, although less pronounced effects were found after exposure to calyculin A.

Another objective was to test whether the addition of phosphatase inhibitors could modulate 8-Cl- and $8\text{-}NH_2$-cAMP induced apoptosis and vice versa. We found no such interaction indicating apoptotic cell death to be induced through separate pathways by 8-Cl-cAMP and phosphatase inhibitors.

*Induction of apoptotic cell death is not associated with specific degradation of nuclear DNA*

Further experiments tested whether nuclear DNA was cleaved specifically during treatment of MCF-7 cells with 8-Cl-cAMP, $8\text{-}NH_2$-cAMP, 8-Cl-adenosine or okadaic acid. There was a lack of massive DNA fragmentation typical of internucleosomal DNA cleavage during the conditions tested (Figures 5 and 6). In experiments designed to test the sensitivity of the DNA assay specific DNA fragments could be detected if 2% of the cells (or 2% of loaded DNA; 0.2 µg of DNA) had specifically degraded chromatin (for details see Figure 6 and Materials and methods section). In detached cells treated with 8-Cl-cAMP and $8\text{-}NH_2$-cAMP a small amount of higher molecular weight DNA smear was noted (Figure 6). Whether this DNA cleavage occurred in the apoptotic or secondary necrotic cells could not be settled by

**Table I** Effect of some selected cAMP analogues, forskolin and IBMX on modulation of growth and induction of cell death in MCF-7 cells

| *Type of addition* | *Number of cells (percentage of reference)* | *+ s.e.m.* | *Number (n)* |
|---|---|---|---|
| 1. Control (5% FCS) | 427 | 14 | 6 |
| 2. 1 μM 8-Cl-cAMP | 432 | 22 | 4 |
| 3. 10 μM 8-Cl-cAMP | 145 | 7 | 10 |
| 4. 31 μM 8-Cl-cAMP | 112 | 14 | 8 |
| 5. 31 μM 8-Cl-cAMP + 2 IU ADA $ml^{-1}$ | 218 | 34 | 3 |
| 6. 31 μM 8-Cl-cAMP + 5% HI-FCS | 325/360 | – | 2 |
| 7. 10 μM 8-$NH_2$-cAMP | 63 | 11 | 4 |
| 8. 31 μM 8-$NH_2$-cAMP | <5 | – | 3 |
| 9. 31 μM 8-$NH_2$-cAMP + 5% HI-FCS | 268/249 | – | 2 |
| 10. 20 μM 8-CPT-cAMP | 410 | 15 | 6 |
| 11. 200 μM 8-CPT-cAMP | 296 | 15 | 9 |
| 12. 500 μM $N^6$-benzoyl-cAMP | 277 | 34 | 3 |
| 13. 20 μM Forskolin | 263 | 25 | 4 |
| 14. 100 μM IBMX | 356 | 14 | 3 |
| 15. 330 μM IBMX | 311 | 27 | 5 |
| 16. 830 μM IBMX | 160 | 15 | 3 |
| 17. 5% FCS + 2 IU $ml^{-1}$ ADA | 395 | 10 | 4 |
| 18. 5% HI-FCS | 438 | 24 | 4 |

The cells were seeded at a density of 12 000 $cm^{-2}$ and grown in DMEM supplemented with 5% FCS (control condition). In conditions 6, 9 and 10 the cells were cultured in 5% heat-inactivated FCS (5% HI-FCS). The cell number was determined after 72 h of treatment, and is expressed as percentage of the cell number determined before the addition of the above agents. Massive cell death was observed in cultures treated with 10 μM 8-Cl-cAMP, 31 μM 8-Cl-cAMP, 10 μM 8-$NH_2$-cAMP, or 30 μM 8-$NH_2$-cAMP (conditions 3,4,7 and 8 respectively) and very little cell death observed in conditions 5,6 and 9. No cell death was observed in conditions 10–18. Standard error of the mean (s.e.m.) and the number (*n*) of separate determinations are listed in columns 3 and 4. ADA, adenosine deaminase; IBMX, 3-isobutyl-1-methylxanthine.

**Figure 2** Test of the reversibility of 8-Cl-cAMP-induced cell death. The cells were supplemented with 100 μM 8-Cl-cAMP (▲) or left unsupplemented as controls. After 8-Cl-cAMP treatment for 7 h (△) or 23 h (□), the cells were washed twice in fresh medium and allowed to continue the incubation in parallel conditioned medium in absence of 8-Cl-cAMP. Each point represents the mean ± s.e.m. of three or more separate experiments (bars) or the mean of two separate experiments (without bars).

**Figure 3** Early morphological effects of 8-$NH_2$-cAMP and 8-Cl-cAMP on attached MCF-7 cells. The cells were exposed to 30 μM 8-$NH_2$-cAMP for 30 h (**b**) or 100 μM 8-Cl-cAMP (**c**) and vehicle (**a**) for 40 h. The cells were fixed and processed for TEM (magnification × 2240).

this method. Also to address the question of whether DNA cleavage might occur in a subpopulation of cells *in situ* nick end-labelling of nuclear chromatin was performed using DIG-dUTP as probe (see Materials and methods section). These studies confirmed that the DNA remained intact in cells treated with okadaic acid (or calyculin A) (Figure 7i and j). However, among cells treated with 8-Cl-cAMP, 8-Cl-adenosine, or 8-$NH_2$-cAMP a considerable fraction was DIG-dUTP positive demonstrating DNA breakage (Figure 7). Cells treated with 8-$NH_2$-cAMP showed preferential DIG-dUTP staining of the marginal chromatin (Figure 7g and h). This was compatible with the morphological pattern of 8-

**Figure 4** Ultrastructure of apoptotic MCF-7 cells. The cells were treated with 100 nM okadaic acid for 12 h (**a**), 30 $\mu$M 8-$NH_2$-cAMP for 30 h (**b**), or 100 $\mu$M 8-Cl-cAMP for 48 h (**c**). The magnifications are × 2300 (**a**,**b**) and × 3200 (**c**). The arrow heads in (**c**) mark the cell border of two partially fused cells.

$NH_2$-cAMP-treated cells showing marginal chromatin condensation in detached cells (Figure 4b). Cells treated with 8-Cl-cAMP showed a more heterogeneous picture. In these cells both condensed and more dispersed chromatin was found among the DIG-dUTP positive cells (Figure 7). The latter phenotype represented most probably secondary necrosis in apoptotic cells although induction of primary necrosis could not be excluded. On closer examination we found both negative and positive condensed nuclei (Figure 7b and d) suggesting these cells to be in different stages of apoptosis; the former perhaps representing an early and the latter a late apoptotic phase, respectively (Figure 7). The DIG-dUTP staining pattern of cells treated with 8-Cl-adenosine closely resembled that of cells treated with 8-Cl-cAMP (Figure 7e and f).

### Discussion

In this report we show that 8-Cl- and 8-$NH_2$-cAMP kill MCF-7 cells by induction of apoptosis through a mechanism that does not involve activation of cAK. More hydrolysis-resistant cAMP analogues, such as 8-CPT-cAMP and $N^6$-benzoyl-cAMP, induced reversible growth inhibition rather than induction of apoptosis in MCF-7 cells (Figure 1, Table I). The effect of these cAMP analogues was mimicked by a microinjected catalytic but not regulatory cAK subunit demonstrating free cAMP to be involved in negative regulation of growth, but not cell death in these cells (Vintermyr *et al.*, 1995)

That 8-Cl-cAMP (and 8-$NH_2$-cAMP) and 8-Cl-adenosine induced cell death by apoptosis rather than necrosis was carefully evaluated. Firstly, all rounded up cells excluded trypan blue, suggesting their plasma membranes to be intact. Secondly, the morphological effects, including an early loss of cell–cell interaction and microvilli, prominent segregation of cellular organelles, marginal or complete condensation of nuclear chromatin, and apparent intactness of mitochondria (Figures 3 and 4) resembled apoptosis (Kerr *et al.*, 1972; Clarke, 1990). Moreover, okadaic acid, previously reported to induce apoptosis in MCF-7 cells (Bøe *et al.*, 1991), induced a similar morphological pattern (Figure 4a). Specific internucleosomal DNA fragmentation was not found during 8-Cl- or 8-$NH_2$-cAMP-induced cell death (Figure 6). In a recent report, 3 days' exposure to low okadaic acid concentrations induced apoptotic cell death in MCF-7 cells in the presence of internucleosomal DNA fragmentation (Kiguichi *et al.*, 1994). We did not find specific DNA fragmentation (Figure 5, insets; Figure 7g and h) in apoptotic MCF-7 cells after exposure to okadaic acid, our results thus being more compatible with those reported by Oberhammer *et al.* on MCF-7 apoptosis induced by serum starvation (Oberhammer *et al.*, 1993). Along with other reports specific internucleosomal DNA fragmentation should not be considered a major criterion of apoptosis (see e.g. Cohen *et al.*, 1992). In cells treated with 8-Cl-cAMP or 8-Cl-adenosine a major fraction of nuclei with condensed morphology (bisbenzimide staining) remained negative with respect to incorporation of DIG-dUTP whereas cells having a more disperse or less condensed chromatin structure became more frequently stained (Figure 7c-f). The results suggest that the initial condensation of nuclear chromatin occurred in the absence of DNA breakage; the latter being a more prominent feature of late apoptotic and secondary necrotic cells. In various fibrosarcoma cell lines a dualism between apoptosis and necrosis was noticed in which the less malignant cells died mainly through apoptosis, whereas the more malignant ones primarily underwent necrosis (Arends *et al.*, 1994). In our study the MCF-7 cells reacted by synchronised induction of apoptosis after addition of okadaic acid suggesting that the triggering of apoptosis at least by this mechanism (Bøe *et al.*, 1991; Jensen *et al.*, 1994) was the same in all MCF-7 cells. Also in MCF-7 implants in nude mice generalised induction of programmed cell death was reported after abrogation of oestrogen (Kyprianou *et al.*, 1991).

Adenosine monophosphate (AMP) is non-toxic and induces reversible inhibition of growth in MCF-7 cells (Hugo *et al.*, 1992). In the present work we show that 8-Cl-adenosine induces apoptotic cell death in MCF-7 cells. A related substance, 2-chloro-2-deoxyadenosine, used for treatment of human cancer, has been reported to induce apoptotic

**Figure 5** Potency of calyculin A (**a**) and okadaic acid (**b**) for rounding of cells and condensation of nuclear chromatin. The cells were treated for 12 h before fixation and staining with bisbenzimide (0.001 mg $ml^{-1}$). The fractions of cells with normal morphology (○,△) and non-condensed nuclear chromatin (●,▲) were determined. The concentrations (doses), giving half maximal deformation of morphology ($ED_{50\,morph}$), were 45 nM for okadaic acid and 3.0 nM for calyculin A. The concentrations (doses), giving half maximal condensation of nuclear chromatin ($ED_{50\,chrom}$), were 188 nM for okadaic acid and 8.9 nM for calyculin A. The results are the mean of parallel samples in two separate experiments. *Insets:* DNA electrophoretic patterns of 10 μg of DNA extracted from MCF-7 cells treated with 50 nM calyculin A (**a**) or 100 nM okadaic acid (**b**) for 20 h.

**Figure 6** Test of internucleosomal DNA fragmentation MCF-7 cells were exposed to 100 μM 8-Cl-cAMP, 30 μM 8-$NH_2$-cAMP, or vehicle (control) for 48 h. DNA was extracted separately from detached (lanes 1 and 2) and adherent (lanes 3–5) cells. An aliquot of 10 μg of DNA was loaded in each lane except for lane 4 in which only 5 μg DNA was loaded since very few cells remained adherent after treatment with 30 μM 8-$NH_2$-cAMP for 48 h (see also Figure 1). The sensitivity for detection of internucleosomal DNA fragments was tested (lanes 6–8) by mixing specifically degraded DNA from apoptotic myeloid leukaemic (IPC-81) cells (Gjertsen *et al*., 1994) with chromosomal DNA from intact (non-apoptotic) primary hepatocytes. Typical DNA ladder patterns are shown after mixing 0.4 μg degraded DNA with 9.6 μg intact chromosomal DNA (lane 6), 1 μg degraded DNA with 9 μg intact chromosomal DNA (lane 7), or 5 μg degraded DNA with 5 μg intact chromosomal DNA (lane 8) alone (for details see Materials and methods section).

cell death in human chronic lymphocytic leukaemia cells (Robertson *et al*., 1993). In a recent study we found that several adenosine analogues at low concentrations were capable of inducing apoptotic cell death in a rat promyeloid leukaemic (IPC-81) cell line; the most potent being 7-Deaza-adenosine (Ruchaud *et al*., 1995). Interestingly, IPC-81 cells, as opposed to MCF-7 cells, were relatively resistant to induction of apoptosis by 8-Cl-adenosine showing that the responsiveness to adenosine analogues may be dependent on cell type. The use of such analogues to provoke apoptotic cell death in pharmacologically less responsive cells, like the MCF-7 cells, could therefore be worth further study. Although the mechanisms of action of adenosine metabolites are not known, both single- or double-stranded breaks in DNA have been reported (Saven *et al*., 1994).

The effect of cAMP-dependent protein kinase is counterbalanced by serine/threonine protein phosphatases (PPs). Specific inhibitors of PPs have proved useful in elucidating the biological effects of these phosphatases (Cohen *et al*., 1990; Mumby and Walter, 1993). In a previous report we found that okadaic acid induced morphological effects resembling apoptosis in several cell types including MCF-7 cells (Bøe *et al*., 1991). Although both okadaic acid and

**Figure 7** Test of chromosomal DNA breakages by the DIG-dUTP nick end-labelling method. The cells were treated with vehicle (**a**,**b**), 100 μM 8-Cl-cAMP (**c**,**d**), 10 μM 8-Cl-adenosine (panels **e**,**f**), 30 μM 8-$NH_2$-cAMP (**g**,**h**) for 48 h, or 100 nM okadaic acid for 20 h (**i**,**j**). Detached cells were pooled by centrifugation and fixed. The structure of nuclear chromatin (**a**,**c**,**e**,**g**, **i**) was visualised by staining with the DNA-specific fluorescent probe bisbenzimide (33258) on rhodamin-anti-DIG-dUTP transferase nick end-labelled (**b**,**d**,**f**,**h**,**j**) specimens.

8-Cl-cAMP (and 8-$NH_2$-cAMP) induced apoptosis in MCF-7 cells, no interaction was found between these agents for induction of apoptosis, suggesting their modes of action to be channelled through separate pathways in the cell. The apoptotic morphology induced by okadaic acid, 8-Cl-cAMP, and 8-$NH_2$-cAMP showed some common features as well as dissimilarities (Figures 3 and 4). That the induction of apoptosis by 8-Cl-cAMP was less synchronised than in the presence of 8-$NH_2$-cAMP could contribute to the difference between these agents. However, heterogeneity in the apoptotic morphology in one single cell type induced by various agents was recently characterised in a leukaemic cell line (Gjertsen *et al*., 1994). One novel feature of cells treated with PP inhibitors was that the morphological effects preceded the condensation of nuclear chromatin (Figure 5). Also, in normal epithelial cells such as primary rat hepatocytes and human keratinocytes, the condensation of nuclear chromatin was a late-onset response to these agents (unpublished results). In some leukaemic cells the condensation of nuclear chromatin could not be separated from the morphological effects occurring in the cytoplasm (Gjertsen *et al*., 1994; Jensen *et al*., 1994), suggesting that the response to PP inhibitors could be different in cells of epithelial and mesenchymal origin.

In this work we show for the first time that 8-Cl-adenosine and easily hydrolysable cAMP analogues like 8-Cl-cAMP and 8-$NH_2$-cAMP induce cell death by apoptosis through a cAMP-independent mechanism. The toxicity of the cAMP analogues was dependent on supplements of serum in the culture media. Conversion of non-toxic cAMP analogues into cytostatic compounds by biodegradation in serum or in tissue could be of pharmacological significance.

**Abbreviations**

cAK, cAMP-dependent protein kinase; 8-Cl-cAMP, 8-chloro-cAMP; 8-Cl-adenosine, 8-chloro-adenosine; 8-$NH_2$-cAMP, 8-amino-cAMP; ADA, adenosine deaminase; IBMX, 3-isobutyl-1-methylxanthine; 8-CPT-cAMP, 8-[4-chlorophenylthio]-cAMP; DIG, digoxigenin.

**Acknowledgements**

The technical support of Nina Lied Larsen is highly appreciated. The work was funded by the Norwegian Cancer Society.

**References**

ALLY S, TORTORA G, CLAIR T, GRIECO D, MERLO G, KATSAROS D, ØGREID D, DØSKELAND SO, JAHNSEN T AND CHO-CHUNG YS. (1988). Selective modulation of protein kinase isozymes by site-selective analog 8-chloroadenosine 3', 5'-cyclic monophosphate provides a biological means for control of human colon cancer cell growth. *Proc. Natl Acad. Sci. USA*., **85**, 6319-6322.

ARENDS MJ, McGREGOR AH AND WYLLIE AH. (1994). Apoptosis is inversely related to necrosis and determines net growth in tumors bearing constitutively expressed myc, ras, and HPV oncogenes. *Am. J. Pathol*., **144**, 1045-1057.

BØE R, GJERSTEN BT, VINTERMYR OK, HOUGE G, LANOTTE M AND DØSKELAND SO. (1991). The protein phosphatase inhibitor okadaic acid induces morphological changes typical of apoptosis in mammalian cells. *Exp. Cell. Res*., **195**, 237-246.

CHO-CHUNG YS, CLAIR T, TORTORA G AND YOKOZAKI H. (1991). Role of site-selective cAMP analogs in the control and reversal of malignancy. *Pharmacol. Ther*., **50**, 1-33.

CHO-CHUNG YS. (1992). Suppression of malignancy targetting cyclic AMP signal transducing proteins. *Biochem. Soc. Trans*., **20**, 425-429.

CLAIR T, ALLY S, TAGLIAFERRI P, ROBINS RK AND CHO-CHUNG YS. (1987). Site selective cAMP analogs induce nuclear translocation of RII cAMP receptor protein in Ha-MuSV-transformed NIH 3T3 cells. *FEBS Lett*., **224**, 377-384.

CLARKE PGH. (1990). Developmental cell death: morphological diversity and multiple mechanisms. *Anat. Embryol*., **181**, 195-213.

COHEN GM, SUN X-M, SNOWDEN RT, DINSDALE D AND SKILLETER DN. (1992). Key morphological features of apoptosis may occur in the absence of internucleosomal DNA fragmentation. *Biochem. J*., **286**, 331-334.

COHEN P, HOLMES CFB AND TSUKITANI Y. (1990). Okadaic acid: a new probe for the study of cellular regulation. *Trends Biochem. Sci*., **15**, 98-102.

DUPREZ E, GJERTSEN BT, BERNARD O, LANOTTE M AND DØSKELAND SO. (1993). Antiapoptotic effect of heterozygously expressed mutant RI (Ala 336→Asp) subunit of cAMP kinase I in a rat leukemic cell line. *J. Biol. Chem*., **268**, 8332-8340.

GAVRIELI Y, SHERMAN Y AND BEN-SASSON SA. (1992). Identification of programmed cell death in situ via specific labelling of nuclear DNA fragmentation. *J. Cell. Biol*., **119**, 493-501.

GJERTSEN BT, CRESSEY LI, RUCHAUD S, HOUGE G, LANOTTE M AND DØSKELAND SO. (1994). Multiple apoptotic death types triggered through activation of separate pathways by cAMP and inhibitors of protein phosphatases in one (IPC leukemia) cell line. *J. Cell Science*, **107**, 3363-3377.

HARTWELL L. (1994). cAMPing out. *Science*, **371**, 286.

HOUGE G, CHO-CHUNG YS AND DØSKELAND SO. (1992). Differential expression of cAMP-kinase subunits is correlated with growth in rat mammary carcinomas and uterus. *Br. J. Cancer*, **66**, 1022-1029.

HUGO F, MAZUREK S, ZANDER U AND EIGENBRODT E. (1992). In vitro effect of extracellular AMP on MCF-7 breast cancer cells: Inhibition of glycolysis and cell proliferation. *J. Cell. Physiol*., **153**, 539-549.

JENSEN PH, CRESSEY LI, GJERTSEN BT, MADSEN P, MELLGREN G, HOKLAND P, GLIMANN J, DØSKELAND SO, LANOTTE M AND VINTERMYR OK. (1994). Cleaved intracellular plasminogen activator inhibitor-2 in human myeloleukemia cells is a marker of apoptosis. *Br. J. Cancer*, **70**, 834-840.

KERR JFR, SEARLE J, HARMON BV AND BISHOP CJ. (1972). Apoptosis: A basic biological phenomenon with wide-ranging implications in tissue kinetics. *Br. J. Cancer*, **26**, 239-257.

KIGUCHI K, GLESNE D, CHUBB CH, FUJIKI H AND HUBERMANN E. (1994). Differential induction of apoptosis in human breast tumour cells by okadaic acid and related inhibitors of protein phosphatases 1 and 2A. *Cell Growth & Differentiation*, **5**, 995-1004.

KYPRIANOU N, ENGLISH HF, DAVIDSON NE AND ISACS JT. (1991). Programmed cell death during regression of the MCF-7 human breast cancer following estrogen ablation. *Cancer Res*., **51**, 162-166.

LANGE-CARTER CA, VUILLEQUES JJ AND MALKINSON AM. (1993). 8-Chloroadenosine mediates 8-chloro-cyclic AMP-induced down-regulation of cyclic AMP-dependent protein kinase in normal and neoplastic mouse lung epithelial cells by a cyclic AMP-independent mechanism. *Cancer Res*., **53**, 393-400.

McCONKEY DJ, ORRENIUS S AND JONDAL M. (1990). Agents that elevate cAMP stimulate DNA fragmentation in thymocytes. *J. Immunol*., **145**, 1227-1230.

MARTI A, JEHN B, COSTELLO E, KEON N, KE G, MARTIN F AND JAGGI R. (1994). Protein kinase A and AP-1 (c-Fos JunD) are induced during apoptosis of mammary epithelial cells. *Oncogene*, **9**, 1213-1223.

MILLER WR, SENBANJO RO, TELFORD J AND WATSON D. (1985). Cyclic AMP binding proteins in human cancer. *Br. J. Cancer*, **52**, 531-535.

MUMBY MC AND WALTER G. (1993). Protein serine threonine phosphatases: structure, regulation, and functions in cell growth. *Physiol. Rev*., **73**, 673-699.

OBERHAMMER F, WILSON JW, DIVE C, MORRIS ID, HICKMAN JA, WAKELING AE, WALKER PR AND SIKORSKA M. (1993). Apoptotic death in epithelial cells: cleavage of DNA to 300 and or 50 kb fragments prior to or in the absence of internucleosomal fragmentation. *EMBO J*., **12**, 3679-3684.

ØGREID D, EKANGER R, SUVA RH, MILLER JP AND DØSKELAND SO. (1989). Comparison of the two classes of binding sites (A and B) of type I and type II cyclic-AMP dependent protein kinases by using cyclic nucleotide analogs. *Eur. J. Biochem*., **181**, 19-31.

ROBERTSON LE, CHUBB S, MEYN RE, STORY M, FORD R, HITTELMAN WN AND PLUNKETT W. (1993). Induction of apoptotic cell death in chronic lymphocytic leukemia by 2-chloro-2'-deoxyadenosine and 9-β-D-arabinosyl-2-fluoroadenine. *Blood*, **81**, 143-150.

ROGER PP, RICKAERT F, HUEZ G, AUTHELET M, HOFMANN F AND DUMONT JE. (1988). Microinjection of catalytic subunit of cyclic AMP-dependent protein kinases triggers acute morphological changes in thyroid epithelial cells. *FEBS Lett*., **232**, 409-413.

ROYCHOUDHURRY R, JAY E AND WU R. (1976). Terminal labelling and addition of homopolymer tracts to duplex DNA fragments by terminal deoxynucleotidyl transferase. *Nucleic Acids Res.*, **3**, 863–877.

RUCHAUD S, ZORN M, DAVILAR-VILLAR E, GENIESER HG, HOFFMANN HG, GJERTSEN BT, DØSKELAND SO, JASTORFF B AND LANOTTE M. (1995). Evidence for several pathways of biological response to hydrolysable cAMP-analogs using a model system of apoptosis in IPC-81 leukemia cells. *Cell. Pharmacol.* (in press).

SAVEN A, LAWRENCE D AND PIRO MD. (1994). 2-chlorodeoxyadenosine: a newer purine analog active in the treatment of indolent lymphoid malignancies. *Ann. Int. Med.*, **120**, 784–791.

VAN LOOKEREN CAMPAGNE MM, DIAZ FV, JASTORFF B AND KESSIN RH. (1991). 8-Chloroadenosine 3', 5'-monophosphate inhibits the growth of Chinese Hamster ovary and molt-4 cells through its adenosine metabolite. *Cancer Res.*, **51**, 1600–1605.

VINTERMYR OK, GJERTSEN BT, LANOTTE M AND DØSKELAND SO. (1993*a*). Microinjected catalytic subunit of cAMP dependent protein kinase induces apoptosis in myeloid leukemia (IPC-81) cells. *Exp. Cell. Res.*, **206**, 157–161.

VINTERMYR OK, BØE R, BRULAND T, HOUGE G AND DØSKELAND SO. (1993*b*). Elevated cAMP gives short term inhibition and long term stimulation of hepatocyte DNA replication. Roles of the cAMP dependent protein kinase subunits. *J. Cell. Physiol.*, **156**, 160–170.

VINTERMYR OK, BØE R, BRUSTUGUN OT, MARONDE E, AAKVAAG A AND DØSKELAND SO. (1995). Cyclic adenosine monophosphate (cAMP) analogs 8-CL- and 8-$NH_2$-cAMP induce cell death independently of cAMP kinase-mediated inhibition of the $G_1$ S transition in mammary carcinoma cells (MCF-7). *Endocrinology*, **136**, 2513–2519.

YOKOZAKI H, TORTORA G, PEPE S, MARONDE E, GENIESER H-G, JASTORFF B AND CHO-CHUNG Y. (1992). Unhydrolysable analogues of adenosine 3':5'-monophosphate demonstrating growth inhibition and differentiation in human cancer cells. *Cancer Res.*, **52**, 2504–2508.